# Influence of Chronic Alcohol Use on Osteoblastic Differentiation of Bone Marrow Cells, Bone Properties, and Hepatic and Renal Morphology of Rats

**DOI:** 10.1155/2018/2494918

**Published:** 2018-07-02

**Authors:** Michelle Cardoso de Sousa, Mariana Raquel da Cruz Vegian, Matheus Alves Biserra, Bruno César Almeida Costa, Samira Esteves Afonso Camargo, Sigmar de Mello Rode, Carolina Judica Ramos, Luciane Dias de Oliveira, Luana Marotta Reis de Vasconcellos

**Affiliations:** ^1^School of Dentistry-Funvic, Faculty of Pindamonhangaba, Braz Cubas University, Mogi das Cruzes, SP, Brazil; ^2^Department of Bioscience and Oral Diagnosis, São Jose dos Campos Institute of Science and Technology, Universidade Estadual Paulista (UNESP), Av. Engenheiro Francisco José Longo, 777, São José dos Campos 12245-000, SP, Brazil; ^3^Department of Prosthodontic and Dental Material, Institute of Science and Technology, Sao Paulo State University (UNESP), Sao Jose dos Campos, SP, Brazil; ^4^Department of Social Dentistry and Children's Clinic, São Jose dos Campos Institute of Science and Technology, Universidade Estadual Paulista (UNESP), Av. Engenheiro Francisco José Longo, 777, São José dos Campos 12245-000, SP, Brazil

## Abstract

Chronic alcohol exposure can affect the osteoblastic activity and the proliferation and differentiation of cells due to its toxic effect, which can affect negatively bone repair and bone microarchitecture. The aim of this study was to evaluate the effects of chronic use of 20% alcohol on rats regarding osteoblastic differentiation, extrinsic and intrinsic properties of the tibia, and hepatic and renal morphology. Wistar rats were divided into three groups (*n* = 9) in accordance with a 24-week diet. After euthanasia, kidneys, liver, and tibias were removed for analysis and femurs mesenchymal cells were collected. The results showed that chronic use of 20% alcohol influenced neither the alkaline phosphatase production nor total protein (*p* > 0.05) in rats, with similar formation of nodules in all groups (*p* > 0.05). However, significant changes in the liver and kidneys and adverse effects on the mechanical properties of the tibia were observed. According to the results, it can be concluded that the chronic use of alcohol for 24 weeks had no negative influence on the activity and differentiation of osteoblasts, but the mechanical properties of the tibia were impaired and the organs responsible for metabolism and excretion were also affected due to the consumption of alcohol.

## 1. Introduction

Due to the toxic effect of alcohol on cells, the bone tissue can be altered as a result of the decrease in osteoblastic activity and cell proliferation and differentiation [1–3]. These effects were observed after direct administration of alcohol into culture cells [1, 2] or those cells of animals ingesting alcohol [3].

The deleterious effect of alcohol can also hit several tissues and systems [4], with the liver being one of the organs most susceptible to the alcohol's toxic effects of [5]. These effects lead to cell injury, which consequently causes severe changes in the hepatic tissue [6–8]. Renal function is also affected by the chronic consumption of alcohol due to the increased metabolization of this substance, consequently causing lesions in the kidney through the formation and excretion of toxic alcohol metabolites [9, 10].

Several factors interfere with the alcohol's action on tissues and cells, such as dose [11] and intake duration [12]. Thus, further studies are needed to evaluate the effects of chronic alcohol consumption and its interference with the maintenance of the physical integrity of living beings. This study aims to evaluate the condition of both hepatic and renal tissues, bone properties and osteoblastic activity in rats with chronic alcohol consumption to understand the cellular mechanisms by which alcohol influences the bone metabolism of these animals.

## 2. Materials and Methods

All the animal procedures were in accordance with the guidelines set by the Animal Research Ethics Committee of the Sao Jose dos Campos Institute of Science and Technology, Sao Paulo State University (UNESP), according to protocol number 003/2010.

### 2.1. Animals

Twenty-seven male Wistar rats of 90 days old with an average body weight of 300 g were kept in cages containing three animals each divided into three groups (*n* = 9) according to treatment with or without alcohol. The animals of the alcohol group (ALC24) were daily treated with 50 ml of 20% alcohol solution (ABS.ACS-99,5o) and 50 g of ration per animal during 24 weeks. The next day, the average consumption of alcoholic solution and ration was recorded. Half portion of food was provided to the animals of the iso-caloric nutritional control group (ISO). This group was treated with liquid diet containing iso-caloric sucrose solution and paired-fed with the same average amount of alcohol and calories ingested by the animals of the ALC group. Control animals (CONT) were treated for 24 weeks ad libitum, and the average amount of feed intake was recorded.

After the treatment period, all animals treated for 24 weeks were anesthetized and sacrificed for cell culture procedures.

### 2.2. Cell Isolation and Primary Culture of Osteogenic Cells

Bone marrow cells obtained from femurs of young adult male Wistar rats submitted to chronic consumption of alcohol or not, as previously described by Maniatopoulos et al., were cultured in *α*-MEM (Gibco, Life Technologies, Grand Island, NY, USA), supplemented with 10% fetal bovine serum (Gibco), 50 mg/ml gentamicin (Gibco), 10^−7^ M dexamethasone (Sigma, St. Louis, MO, USA), 5 mg/l ascorbic acid (Gibco), and 2.16 g/l a-glycerophosphate (Sigma). Next, the cells were cultured in 24 microplate wells and incubated at 37 with 5% CO_2_. The cells also were cultured on titanium samples with sandblasted surface. The osteoblasts were characterized for different analysis after 3 and 7 days of culture [13]. All tests were performed in triplicate.

#### 2.2.1. Tests for Activity Cellular

Total protein content and alkaline phosphatase activity (ALP) were determined after 3 and 7 days of culture. Both procedures for these analyses were described by De Andrade et al. and Prado et al. [14, 15].

The analysis of the mineralized matrix nodules were measured after 14 days of culture. The cells were incubated at 37°C and 5% CO_2_ in bottom microplate and on titanium samples. The formation of mineralized nodules was quantified by using staining with 2% Alizarin red S (Sigma Chemical, USA) and measured on both surfaces with a spectrophotometer operating at 405 nm according to De Andrade et al. and Prado et al. [14, 15]. The values were described as absorbance.

### 2.3. Histological Analysis of Liver and Kidneys

For histological evaluation, right kidney and liver were removed and fixed with 10% formalin. Next, they were embedded in paraffin and five sections of five-micrometer thick were obtained semiserially per specimen. The sections were stained with hematoxylin and eosin (H&E) for analysis of their morphology.

### 2.4. Analysis of the Intrinsic and Extrinsic of Mechanical Properties of the Tibia

After euthanasia, the tibias were removed and preserved in Ringer's solution (Sanobiol, Pouso Alegre, MG, Brazil) at −20°C until being submitted to tests at room temperature. The extrinsic properties of the tibia were assessed at three points of flexural test, which measured the strength and stiffness of the tibia by means of a universal testing machine (Emic®, model DL 200 MF, São José dos Pinhais, Brazil). The tibias were placed in the holder and a 50-kgf load was applied with a cylinder of 3.14 mm in diameter at a 0.5 mm/min cross-head speed until peak load was achieved (fracture). The specimens were kept moistened with 0.9% saline solution (Sanobiol, Pouso Alegre, MG, Brazil) throughout the testing procedures. After the fracture, the longest and shortest distances between external and internal cortical bones were assessed by using the Image J software version 1.46. The measurements found were then used for calculating the maximum strength and elasticity modulus (i.e., intrinsic properties), according to the ANSI/ASABE S459, Mar 1992 (R2007).

### 2.5. Statistical Analysis

The* in vitro *data and extrinsic properties were submitted to two-way analysis of variance (ANOVA) followed by Tukey's test, which was used for multiple comparisons (GraphPad Prism software, v. 5.01). Data on intrinsic properties were submitted to Kruskal-Wallis test followed by Mann–Whitney test, also for multiple comparisons. *p* < 0.05 was considered statistically significant.

## 3. Results

### 3.1. Weight of Animals

All animals presented weight gain during the treatment period, but ISO group showed no statistically significant difference in the values found. When analyzing the mean in percentage of the weight gain among the groups, it was verified that the control group presented a higher percentage of weight gain, with statistically difference for the two experimental groups (ALC *p* < 0,05 and ISO *p* < 0,001) (Table 1).

It was observed that the control group presented a higher mean (*p* < 0.001) of solid diet intake compared to the experimental group. Regarding the average fluid consumption of the ALC and ISO groups, it was verified that the animals of the ISO group consumed less sucrose solution when compared to the alcohol intake by the ALC group. This difference was statistically significant (*p* < 0,05) (Table 1).

Table 2 shows a more detailed analysis of the nutritional consumption of the animals in the alcohol group. It was observed that 45% of the total calorie consumed by the animal from the ALC group came from the alcoholic diet.

Comparing the ALC group with the ISO group, both fed daily with the same amount of feed; it means the same caloric amount of solid diet. Regarding the liquid diet, the ALC group consumed a higher quantity of calories (30.24 Kcal/day/rat) compared to the ISO group (27.89 Kcal/day/rat). CONT group, during the same period of experiment, received ration and water at will, which generated a higher Kcal consumption by these animals.

### 3.2. Total Protein Content

**Figure 1:**
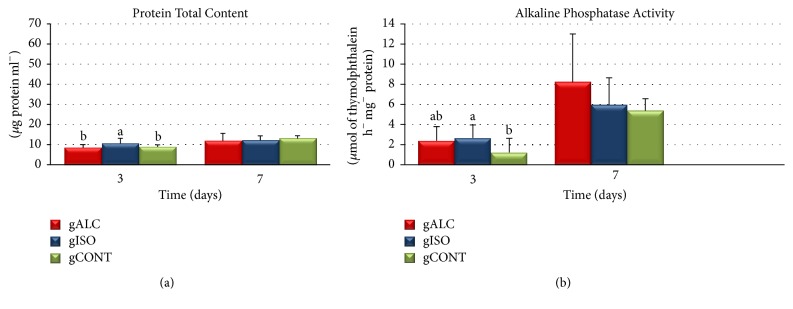
Means and standard deviations of* in vitro *tests for each experimental group after 3 and 7 days of culture: (a) total protein content and (b) alkaline phosphatase activity.

The total protein content increased as a function of time, being greater in the control group (Figure 1). At day 3, no significant difference was observed between ALC (8.50 ± 1.51 mg/mL) and CONT (8.4 ± 1.03 mg/mL) groups. However, the ISO group (10.61 ± 2.44 mg mL) had a statistically higher mean value than the others (*p* < 0.05). At day 7, although the CONT group showed the highest values (13.03 ± 1.38 mg/mL), they were not statistically different (*p* > 0.05) compared to those of ALC (11.74 ± 3.86 mg/mL) and ISO (12.00 ± 2.30 mg/mL) groups.

### 3.3. Alkaline Phosphatase Activity

In all groups, the higher production of ALP was observed within 7 days (Figure 1). At day 3, the ALC group showed higher values compared to those of CONT group, but lower than those of ISO group. There was a statistically significant difference between ISO and CONT groups (*p* < 0.05). At day 7, no statistically significant difference was found between the groups (*p* > 0.05).

### 3.4. Formation of Mineralized Nodule

**Figure 2:**
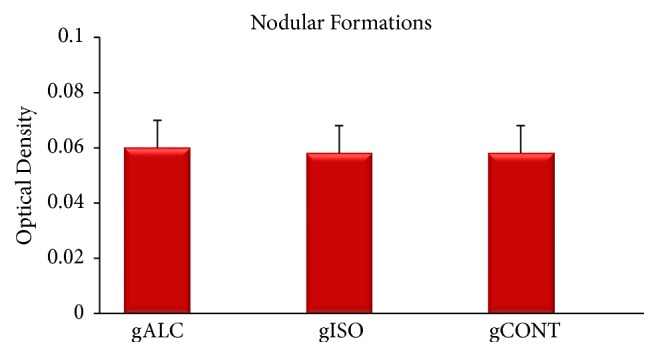
Mean values, in optical density, of nodular formations in plate background after 14 days of culture in the ALC, ISO, and CONT groups.

All the groups presented statistically similar values (*p* = 0.87) (Figure 2). As for the formation of nodules at the bottom of the microplate, the mean and standard deviation values in the groups were the following: ALC24 (0.060 ± 0.01), ISO24 (0.058 ± 0.01), and CONT24 (0.058 ± 0.01). All groups showed similar results in the microplate background, with no difference (*p* > 0.05) between the groups.

Regarding the nodular formation on the surface of blasted titanium samples, no statistically significant difference was observed among the ALC, ISO, and CONT groups. ALC (0.055 ± 0.01) had a mean value similar to CONT (0.055 ± 0.01) but was slightly higher than ISO (0.054 ± 0.01) (*p* = 0.98).

### 3.5. Descriptive Histological Analysis of the Liver

**Figure 3:**
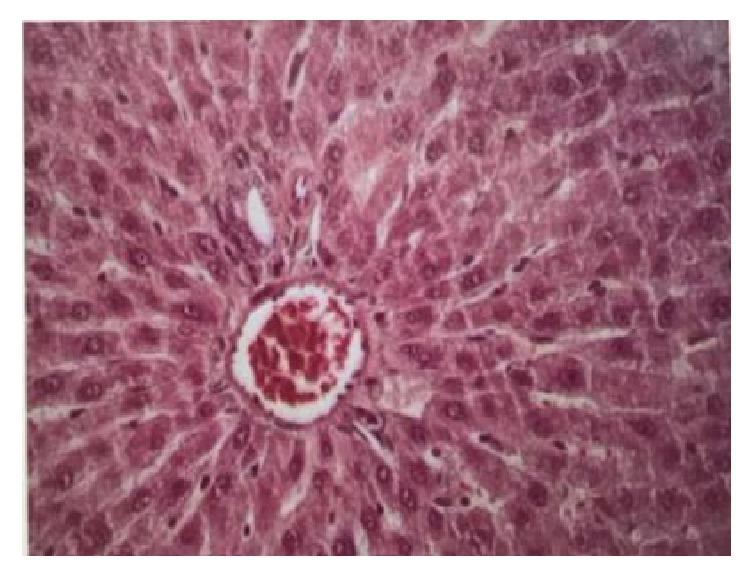
Door space in hepatic tissue. Trabeculae of normal hepatocytes interspersed with sinusoidal capillaries. Control group, HE. 400x.

**Figure 4:**
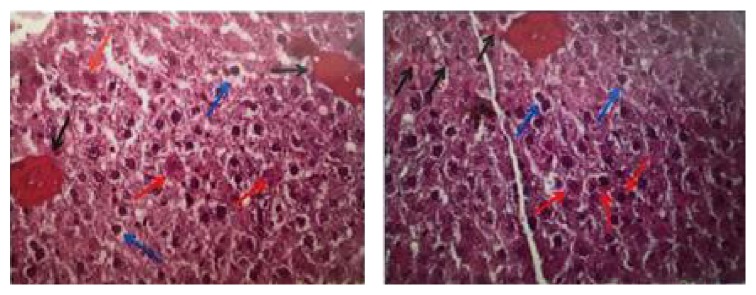
Histological changes of the liver: (blue arrow) hepatocytes with hydropic degeneration; (red arrow) hepatocytes with hyaline degeneration (Mallory corpuscles); (black arrow) pyknotic nuclei. Alcohol group, HE 400x.

In the control group, the hepatic tissue showed trabeculae containing polygonal hepatocytes with one or two large nuclei centrally located in the cells with homogeneous cytoplasm interwoven among sinusoidal capillaries (Figure 3). In the alcohol group, on the other hand, the hepatocytes showed accumulation of fat in the cytoplasm as small droplets, either isolated or associated with form macrovesicles. It was also possible to observe various degrees of hydropic tumefaction of hepatic cells, mainly in the central-lobular region, where cell morphological changes occur as polygonal cells shift to balloon-shaped ones. The presence of cells with pyknotic nuclei and hepatocytes with perinuclear eosinophilic material, giving origin to Mallory's corpuscles, was observed as well as deposition of hyaline material obliterating veins and sinusoids (Figure 4). In the iso-caloric group, trabeculae with polygonal hepatocytes with centrally located large nuclei were also found.

### 3.6. Descriptive Histological Analysis of the Kidneys

**Figure 5:**
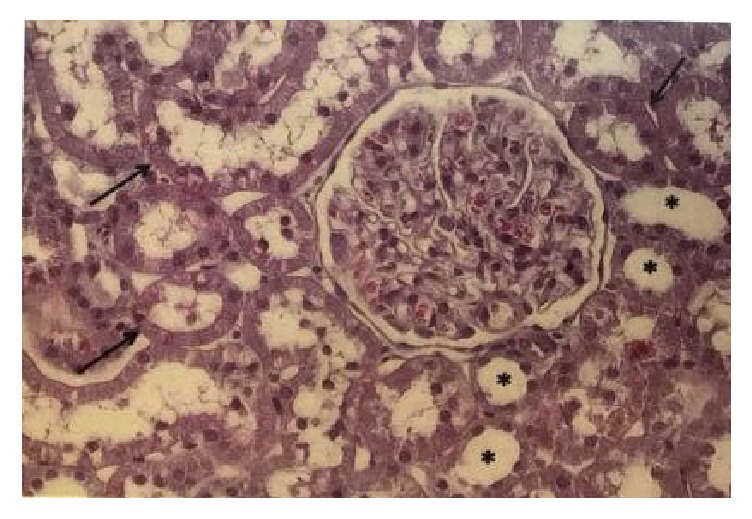
Histology of renal tissue. Glomeruli with normal aspect: (black arrow) proximal convoluted tubules. ^*∗*^Distal convoluted tubules. Control group, HE 400x.

In the control group, histological sections showed glomeruli with corpuscles containing capillaries, mesangial cells, and normal matrix, including Bowman's space. Distal convoluted tubules exhibiting flat cuboidal cells with moderate acidophilia and large nuclei were observed, whereas other cuboidal cells exhibited proximal convoluted tubules, centrally located large nuclei and microvilli, thus characterizing a brush-shaped surface (Figure 5).

**Figure 6:**
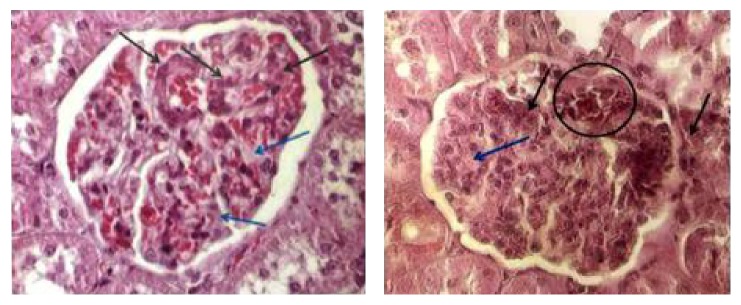
Histological changes in renal glomeruli: (black arrow) proliferation of mesangial cells; (blue arrow) increase in the amount of intercellular matrix. Dilatation of capillaries with hyperemia. Capillary circle extensively dilated with blood cells. Alcohol group. HE 400x.

**Figure 7:**
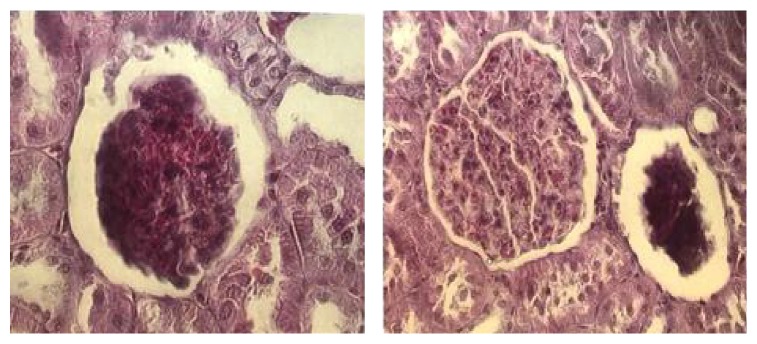
Histological changes in renal glomeruli. Two glomeruli with complete loss of their normal structure, presenting agglomerated cells, congested capillaries. Alcohol group. HE 400x.

The animals of group ALC showed proliferation of mesangial cells, increased amount of matrix and capillary dilatation with presence of hyperemia (Figure 6). In addition, due to the proliferation of these cells, some glomeruli were found to be larger, with decreased Bowman's space. There were also glomeruli exhibiting loss of histological characteristics, with masses of bundled cells and congested capillaries with consequent decrease in the volume of such structures (Figure 7). Regions showing intense chronic inflammatory process were found, with predominance of lymphocytes. Such an occurrence altered the tissue structure, resulting in disorganization of the duct arrangement in the region and structural changes in the glomeruli. In addition, hyaline and eosinophilic cylinders were found to obliterate the renal tubules, which were dilated.

Analysis of histological sections obtained from animals of the iso-caloric group exhibited normal glomeruli, but tubular dilatation and atrophy of these structures were observed.

### 3.7. Intrinsic and Extrinsic Mechanical Properties of the Tibia

Data on extrinsic (i.e., maximum force and rigidity) and intrinsic (i.e., maximum strength and elasticity modulus) mechanical properties are shown in Table 3.

The ALC group had the lowest values of maximum force and rigidity, with statistically significant difference (*p* < 0.05) compared to ISO group, whereas the control group showed no such difference compared to the other groups (*p* > 0.05). The intrinsic properties of the tibia, represented by maximum strength and modulus of elasticity, were also found to be lower in the ALC group compared to CONT and ISO groups, differing statistically from them (*p* < 0.01).

## 4. Discussion

Chronic ingestion of alcohol can affect the osteoblastic activity and change the synthesis of proteins [16]. In the process of osteoblastic differentiation from mesenchymal cells of bone marrow, there are expressions of several proteins [17], such as alkaline phosphatase, which has been evaluated by various* in vitro *studies as an osteoblastic differentiation factor [16, 18]. In the present study, the metabolic activity of osteoblasts was assessed and both content of total protein and activity of alkaline phosphatase did not exhibit any statistical differences between CONT and ALC groups, regardless of the experimental period group of time. Nevertheless, group ISO differed from the others at day 3. Some authors found a decreased activity of alkaline phosphatase when cell cultures were directly exposed to alcohol [19–21]. However, studies on animals ingesting alcohol demonstrated an increased activity of this enzyme [3, 22], whose results are similar to those found in the present study. There is evidence of decreased osteoblastic activity when cell culture is directly* in vitro *exposed to alcohol during 24 hours [23]. On the other hand, Rosa et al. studied cells from animals ingesting alcohol and reported results similar to ours [3].

The high enzymatic production by osteoblasts is related to deposition of calcium [16].* In vitro *evaluation of the formation of mineralization nodules is considered a key parameter in the analysis of the mineralization of extracellular matrix secreted by these cells, which reflects in the cell differentiation [24]. In this study, there was no statistically significant difference between the groups regarding the formation of mineralized matrix. Again, one can observe that previous studies reported different results regarding the correlation between formation of mineralized matrix and effect of alcohol. Hipp et al. exposed osteoblastic cells to alcohol and reported an increased production of mineralized matrix nodules, a finding different from ours [22].

As for* in vivo *studies, factors which may overestimate the effects of alcohol on bone loss include intrinsic variables in specimens or subjects, in addition to doses, intake time, and abstinence of alcohol. There are many differences in the histomorphometric and biomechanical measurements of the effect of alcohol on the activity of bone cells, mainly when alcohol intake is increased, since these animals have a quick growth and time of intake influences the differentials of bone loss characteristics [25]. Therefore, in* in vivo *studies, statistically significant differences may not be found for prolonged times of alcohol ingestion. However, it is possible to suppose that* in vitro *studies may show that cells can resume their functions in the absence of alcohol, which is very interesting in cases when patients stops ingesting this substance, although further clinical studies are necessary to confirm this possibility.

Another fact related to alcohol is its capacity to change negatively the quality of bone mechanical properties by decreasing them [26, 27], and this study has also evaluated the intrinsic and extrinsic mechanical properties of the tibia in animals by measuring bone deformation before fracture and bone strength to load. The results indicated high coefficients of intragroup variation for rigidity, regardless of the treatment. However, although the ALC group showed statistically smaller values of deformation compared to ISO group, the same was not observed in relation to the CONT group, a finding also observed by Hogan et al. after 6–12 months of treatment [26]. As for the intrinsic mechanical properties, it was possible to find statistically lower values in the ALC group compared to CONT group in both studies, demonstrating that alcohol has a negative effect on tibia.

Therefore, in the present study, it was observed that alcohol consumption contributed negatively to the mechanical properties intrinsic to the tibia. These properties reflect on the organic and inorganic quality of the bone, as well as its response to remodeling. It is observed that chronic alcohol consumption negatively affects osteoblast differentiation and activity and contributes to the differentiation and activity of osteoclasts. In addition, it alters the organization of plasma cell membranes, impairing the regulatory pathways of bone cells. All these factors generate unsatisfactory impacts on bone metabolism [28].

In addition, histological characterization of the liver and kidneys can also reveal important changes which may serve to evaluate the negative effects of the chronic use of alcohol. Liver and kidneys are the main organs accounting for metabolization of alcohol and excretion of metabolites, respectively, thus being extremely affected by its intake [5]. In the present study, the main change in the hepatic tissue was the baloonization of hepatocytes in the ALC and ISO groups as a result of their vacuolar degeneration, a feature reported in the cases of alcoholic hepatitis. In the ALC group, balloon-shaped cells were observed across the whole liver, as well as other changes such as presence of eosinophilic material around the hepatocytes, presence of tubules filled with amorphous hyaline material, and congested blood vessels, resulting in normal loss of tissue structure. These changes were also found by other authors, with lesion extension in the hepatic tissue depending on the time of alcohol ingestion by the animals [8, 29, 30].

The renal tissue presented more histological changes in the ALC group compared to ISO and CONT groups. Renal glomeruli were found to be extensively changed, exhibiting different characteristics from those found in normal situations, as observed in the CONT group. These changes were also observed by other authors following administration of alcohol [8, 31]. Many glomeruli showed reduced Bowman's space, structural atrophy, and agglomeration of mesangial cells, a finding also reported by studies using a protocol of 1–8-week ingestion of alcohol [8, 31]. As the majority of the works performed treatments shorter than ours (24 weeks), it is thought that the longer the time of alcohol administration, the more intense the changes in these tissues.

## 5. Conclusion

According to the results found, one can conclude that the chronic use of 20% alcohol in rats over a period of 24 weeks has not negatively influenced the activity and differentiation of osteoblasts, suggesting a recovery of the cell functioning in the absence of alcohol. Nevertheless, the mechanical properties of the tibia were impaired due to the consumption of alcohol. In addition, the organs accounting for metabolization and excretion were also widely affected.

## Figures and Tables

**Table 1:** Mean and standard-deviation values of food, alcohol, and sucrose solution intake.

Weight gain%	Average ± standard deviation	
ALC	15,62% ± 7,37%	A
ISO	4,69% ± 7,81%	B
CONT	23,88% ± 7,70%	C

Solid Diet (g/dia/Rat)	Average ± standard deviation	

ALC	15.49 ± 1.64	A
ISO	15.49 ± 1.64	A
CONT	25.83 ± 2.66	B

Liquid Diet (ml/dia/Rat)	Average ± standard deviation	

ALC	27.09 ± 3.28	A
CONT	24.99 ± 2.14	B

Different letters indicate statistically significant difference.

**Table 2:** Analysis of alcohol consumption and animal ration of ALC group.

Alcohol consumption	Values
Mean intake of alcohol solution 20% (ml/day/rat)	27,09 ml
Mean intake of absolute alcohol (ml/day/rat)	5,42 ml
Mean intake of absolute alcohol (g/day/rat)	4,26 g
Mean intake of absolute alcohol (g/kg weight/dia)	8,87 g
Intake of alcohol (Kcal/day/rat)	30,24 Kcal

Ration intake	Values

Mean intake of ration (g/day/rat)	15,49 g
Mean intake of ration (Kcal/day/rat)	36,95 Kcal
% caloric intake from alcoholic diet (mean of day/rat)	45%

**Table 3:** Mean and standard-deviation values of intrinsic and extrinsic mechanical properties of the tibia.

Groups	ALC	ISO	CONT
Extrinsic properties			

Maximum force	108,8 ± 5,0 N^a^	117,4 ± 14,7 N^b^	115,1 ± 14,6 N^ab^
Rigidity	236,3 ± 19,6 N/mm^a^	268,5 ± 34,3 N/mm^b^	246,3 ± 45,1 N/mm^ab^

Intrinsic properties			

Maximum strength	2,42 ± 2,7 Pa^a^	5,03 ± 3,5 Pa^b^	4,16 ± 3,7 Pa^bc^
Elasticity modulus	1,81 ± 0,3 Pa^a^	3,54 ± 2,7 Pa^b^	2,67 ± 1,11 Pa^bc^

Different letters indicate statistically significant difference.

## References

[1] Klein R. F., Fausti K. A., Carlos A. S. (2003). Ethanol inhibits human osteoblastic cell proliferation. *Alcoholism: Clinical and Experimental Research*.

[2] Vignesh R. C., Sitta Djody S., Jayasudha E. (2006). Effect of ethanol on human osteosarcoma cell proliferatation, differentiation and mineralization. *Toxicology*.

[3] Rosa M. L., Beloti M. M., Prando N., Queiroz R. H. C., de Oliveira P. T., Rosa A. L. (2008). Chronic ethanol intake inhibits in vitro osteogenesis induced by osteoblasts differentiated from stem cells. *Journal of Applied Toxicology*.

[4] Aroor A. R., Shukla S. D. (2004). MAP kinase signaling in diverse effects of ethanol. *Life Sciences*.

[5] Pina A., Morgado S. (2007). Etanol, membranas celulares, alcoolismo. *Actas Bioq*.

[6] Junior A. A. J., Meirelles M. S., Chiarello P. G., Vannucchi H. (2002). Efeito da administração crônica de etanol sobre a peroxidação lipídica em ratos. *Medicina*.

[7] Díaz Gómez M., Fanelli S., delgado de Layño A., Bietto F., Castro J., Castro G. (2008). Deleterious effects induced by oxidative stress in liver nuclei from rats receiving an alcohol-containing liquid diet. *Toxicology & Industrial Health*.

[8] Rocha S. W. S., Silva B. S., Gomes F. O. D. S. (2012). Effect of diethylcarbamazine on chronic hepatic inflammation induced by alcohol in C57BL/6 mice. *European Journal of Pharmacology*.

[9] Epstein M. (1997). Alcohol′s impact on kidney function. *Alcohol Health Res World*.

[10] De Oliveira A. S., Kersul A. P., Prado J. P., Oliveira J. A., Soares E. A. (2011). Efeitos do alcoolismo crônico na morfologia renal de ratos Wistar. *Review of Brazilian Clinical Medicine Journal*.

[11] de Souza D. M., Ricardo L. H., Kantoski K. Z., da Rocha R. F. (2009). Influence of alcohol consumption on alveolar bone level associated with ligature-induced periodontitis in rats. *Brazilian Oral Research*.

[12] Sripanyakorn S., Jugdaohsingh R., Mander A., Davidson S. L., Thompson R. P. H., Powell J. J. (2009). Moderate ingestion of alcohol is associated with acute ethanol-induced suppression of circulating CTX in a PTH-independent fashion. *Journal of Bone and Mineral Research*.

[13] Maniatopoulos C., Sodek J., Melcher A. H. (1988). Bone formation in vitro by stromal cells obtained from bone marrow of young adult rats. *Cell and Tissue Research*.

[14] De Andrade D. P., De Vasconcellos L. M. R., Silva Carvalho I. C. (2015). Titanium-35niobium alloy as a potential material for biomedical implants: In vitro study. *Materials Science and Engineering C: Materials for Biological Applications*.

[15] Prado R. F., de Oliveira F. S., Nascimento R. D., de Vasconcellos L. M., Carvalho Y. R., Cairo C. A. (2015). Osteoblast response to porous titanium and biomimetic surface: in vitro analysis. *Materials Science & Engineering C: Materials for Biological Applications*.

[16] Alves R. D. A. M., Eijken M., Swagemakers S. (2010). Proteomic analysis of human osteoblastic cells: Relevant proteins and functional categories for differentiation. *Journal of Proteome Research*.

[17] Okamura H. (2017). Role of protein phosphatase 2A in osteoblast differentiation and function. *Journal of Clinical Medicine*.

[18] Mathews S., Bhonde R., Gupta P. K., Totey S. (2012). Extracellular matrix protein mediated regulation of the osteoblast differentiation of bone marrow derived human mesenchymal stem cells. *Differentiation*.

[19] Wezeman F. H., Gong Z. (2004). Adipogenic effect of alcohol on human bone marrow - Derived mesenchymal stem cells. *Alcoholism: Clinical and Experimental Research*.

[20] Torricelli P., Fini M., Giavaresi G. (2008). Chronic alcohol abuse and endosseous implants: linkage of in vitro osteoblast dysfunction to titanium osseointegration rate. *Toxicology*.

[21] Maran A., Zhang M., Spelsberg T. C., Turner R. T. (2001). The dose-response effects of ethanol on the human fetal osteoblastic cell line. *Journal of Bone and Mineral Research*.

[22] Hipp J. A., Hipp J. D., Atala A., Soker S. (2010). Ethanol alters the osteogenic differentiation of amniotic fluid-derived stem cells. *Alcoholism: Clinical and Experimental Research*.

[23] Li J., Zhang F.-Q., Du Z.-N., Cai T., Cai P.-S., Fan L. (2015). Protective effect of HO-1 transfection against ethanol-induced osteoblast damage. *Journal of Huazhong University of Science and Technology (Medical Sciences)*.

[24] Hoemann C. D., El-Gabalawy H., McKee M. D. (2009). *In vitro* osteogenesis assays: influence of the primary cell source on alkaline phosphatase activity and mineralization. *Pathologie Biologie*.

[25] Chakkalakal D. A. (2005). Alcohol-induced bone loss and deficient bone repair. *Alcoholism: Clinical and Experimental Research*.

[26] Hogan H. A., Groves J. A., Sampson H. W. (1999). Long-term alcohol consumption in the rat affects femur cross-sectional geometry and bone tissue material properties. *Alcoholism: Clinical and Experimental Research*.

[27] Hogan H. A., Argueta F., Moe L., Nguyen L. P., Sampson H. W. (2001). Adult-onset alcohol consumption induces osteopenia in female rats. *Alcoholism: Clinical and Experimental Research*.

[28] Gaddini G. W., Turner R. T., Grant K. A., Iwaniec U. T. (2016). Alcohol: a simple nutrient with complex actions on bone in the adult skeleton. *Alcoholism: Clinical and Experimental Research*.

[29] Acharya S., Mehta K., Rodriguez S., Pereira J., Krishnan S., Rao C. V. (1997). A histopathological study of liver and kidney in male Wistar rats treated with subtoxic doses of t-butyl alcohol and trichloroacetic acid. *Experimental and Toxicologic Pathology*.

[30] Flora S. J. S., Pant S. C., Malhotra P. R., Kannan G. M. (1997). Biochemical and histopathological changes in arsenic-intoxicated rats coexposed to ethanol. *Alcohol*.

[31] Borole K., Bodhankar S., Dawane J., Kanwal J. K. (2012). Hepatorenal repercussions of alcoholic exposure in a rat model: a dose-dependent study of metformin intervention. *The Iranian Biomedical Journal*.

